# An In Vitro Study on the Antibacterial Activity of Statins Against the Most Common Bacterial Pathogens Causing Respiratory Tract Infections in a Tertiary Care Hospital

**DOI:** 10.7759/cureus.58108

**Published:** 2024-04-12

**Authors:** Preetha Selva, Sheela Durairajan

**Affiliations:** 1 Pharmacology, Saveetha Medical College and Hospital, Saveetha Institute of Medical and Technical Sciences, Saveetha University, Chennai, IND

**Keywords:** pleiotropic, minimum inhibitory concentration, zone of inhibition, respiratory tract infections, antibacterial, statins

## Abstract

Introduction

The vast pleiotropic effect of statins has intrigued many researchers to select them as potential candidates against bacterial infections. The role of statins against bacterial pathogens remains debatable. This study was undertaken to evaluate and compare the antibacterial effect of commonly available statins against the most frequently isolated bacterial pathogens causing respiratory tract infections in our tertiary care hospital using sputum as a sample.

Materials and methods

The study was conducted in the Microbiology Laboratory of our hospital. Drugs including atorvastatin, rosuvastatin, and simvastatin were purchased in pure form from Sigma Aldrich. Dimethylsulfoxide (DMSO) was used as a solvent for all three drugs. The positive controls used were gentamycin and amoxicillin for Gram-negative and Gram-positive bacteria, respectively.

Data regarding all the culture and sensitivity results of sputum samples of patients admitted to the Respiratory Intensive Care Unit over the past 12 months were analyzed. The most common bacterial pathogens *Staphylococcus aureus, Klebsiella pneumoniae, *and *Streptococcus pneumoniae* isolated from sputum specimens were taken for our study.

The antibacterial effect of statins was studied using two methods: the agar cup diffusion method and the broth dilution method. The zone of inhibition and minimum inhibitory concentration of the drugs were calculated and analyzed. Statistical analysis was performed using GraphPad Prism software version 10.2.0. A one-way ANOVA test was used to determine if there was any statistical difference between the different statins and antibiotic groups. An unpaired t-test was used to determine the statistical difference between the statins.

Results and discussion

For the agar cup diffusion method, our results displayed a lack of antibacterial activity of all three statins atorvastatin, rosuvastatin, and simvastatin against all three bacterial strains *Staphylococcus aureus, Klebsiella pneumoniae, *and *Streptococcus pneumoniae* after overnight incubation by agar cup method at concentrations of 3.125 μg/ml, 6.25 μg/ml, 12.5 μg/ml, 25 μg/ml and 50 μg/ml, respectively. The zone of inhibition observed was less than 4 mm (resistant) for all the serial dilutions of atorvastatin, rosuvastatin, and simvastatin.

For the broth dilution method, the ANOVA test showed amoxicillin and gentamicin to have high statistically significant microbial growth inhibitory activity (p-value < 0.005) compared to atorvastatin and rosuvastatin. Statistically, though atorvastatin showed significant antimicrobial activity compared to normal saline and rosuvastatin, this was not considered clinically significant as the antimicrobial activity shown by atorvastatin was very negligible compared to the controls used and did not correspond to the serial dilutions of the drug.

Conclusion

Atorvastatin, rosuvastatin, and simvastatin lacked antibacterial activity against all three bacterial strains isolated from sputum specimens: *Staphylococcus aureus, Klebsiella pneumoniae,* and *Streptococcus pneumoniae*. Hence, the use of statins as an antimicrobial drug for respiratory tract infections has limited applications.

## Introduction

Statins have been widely used for many decades in patients with dyslipidemia. It is considered the gold standard drug among medical practitioners as it has shown promising benefits in reducing cardiovascular mortality and stroke despite lowering lipid levels [1, 2]. The most commonly used statins available in India are atorvastatin, rosuvastatin, and simvastatin [3]. Statins primarily act by inhibiting hydroxymethylglutaryl-coenzyme A (HMG-CoA) reductase, an enzyme needed for the biosynthesis of cholesterol. As a compensatory mechanism, there is an upregulation of hepatic LDL receptors, resulting in the clearance of LDL cholesterol levels in the circulation. However, the cardioprotective role demonstrated by statins is independent of their LDL-lowering mechanism. Many studies have been done so far on these pleiotropic effects of statins [4-7]. The known pleiotropic effects of statins include atheromatous plaque stabilization, enhanced endothelial functioning, anti-inflammatory effects, reduction of oxidative stress, etc. These beneficial effects of statins have instigated researchers across the globe to explore the other pleiotropic effects of statins. Many studies have been done to detect the efficacy of statins in bone metabolism and osteoporosis, including the antitumor effect, immunomodulatory effect, anticoagulant effect, antiaging effect, anti-ischemic effect, antiarrhythmic effect, antibacterial, antiviral, antifungal effects, and so forth.

India, still being a developing country, suffers from a superfluous increase in infectious diseases [8,9]. Judicious and rational use of antibiotics has been the mainstay of treatment for bacterial infections. Emerging antibiotic resistance and an increased incidence of side effects are major drawbacks of using antibiotics. Hence, there is a quest for newer antimicrobials to overcome these problems. Repurposing of existing drugs has become more sought-after as it cuts the drug development timeline and costs associated with it. The vast pleiotropic effect of statins has intrigued many researchers to select them as potential candidates against bacterial infections. Many studies have shown the effect of different statins against bacterial pathogens causing skin infections, urinary tract infections, and sepsis [10-14]. Very few studies have also been done on the antibacterial effect of statins on pathogens causing respiratory tract infections using blood as a sample [15]. The most widely accepted, noninvasive, and optimal sample for the diagnosis of bacterial pathogens causing respiratory tract infections is, however, the sputum [16]. No study has been done so far on bacterial pathogens causing respiratory tract infections using sputum as a sample.

Moreover, there are also contradictory studies regarding the antibacterial effect of statins. Hence, the role of statins against bacterial pathogens remains debatable.

Owing to the aforementioned limitations and gaps of the previous studies, this study was undertaken to evaluate and compare the antibacterial effect of commonly available statins against the most frequently isolated bacterial pathogens causing respiratory tract infections in our tertiary care hospital, using sputum as a sample.

## Materials and methods

Study design, duration, and place

This in vitro study was conducted for a period of three months, from September 2023 to November 2023, in the Microbiology Laboratory of Saveetha Medical College, Saveetha Institute of Medical and Technical Sciences.

Chemicals used

Drugs atorvastatin, rosuvastatin, and simvastatin were purchased in pure form from Sigma Aldrich. Dimethylsulfoxide (DMSO) was used as a solvent for all three drugs. The positive controls used were gentamycin and amoxicillin for gram-negative and gram-positive bacteria, respectively.

Procedure

The study was conducted after getting approval from the Institutional Ethics Committee (approval number: 126/03/2024/Faculty/SRB/SMCH) and the head of the Microbiology Department. Data regarding all the culture and sensitivity results of sputum samples of patients admitted to the Respiratory Intensive Care Unit over the past 12 months were analyzed. The most common bacterial pathogens, *Staphylococcus aureus, Klebsiella pneumoniae, *and* Streptococcus pneumoniae*, were isolated from sputum specimens for our study. The antibacterial effect of statins was studied using two methods: the agar cup diffusion method and the broth dilution method.

Antibacterial effect of statins using the agar cup diffusion method

The bacterial strains (*Staphylococcus aureus, Klebsiella pneumoniae*, and *Streptococcus pneumoniae*) were isolated from sputum specimens. DMSO was used as a solvent for our study as it has no antibacterial activity of its own. Gentamycin was used as a positive control for gram-negative bacteria like *Klebsiella pneumoniae*, and amoxicillin was used as a positive control for gram-positive bacteria like S*taphylococcus aureus *and *Streptococcus pneumoniae*. A stock solution of 100 mcg/ml was prepared for atorvastatin, rosuvastatin, and simvastatin. Serial dilutions of 3.125 μg/ml, 6.25 μg/ml, 12.5 μg/ml, 25 μg/ml, and 50 μg/ml were prepared for each of the three drugs. We have restricted the concentration to a maximum of 50 ug/ml, as the normal human serum concentration of statins at therapeutic doses is only 1-15 nmol/l [17]. The test agar plate was swabbed with the bacterial pathogens* Staphylococcus aureus, Klebsiella pneumoniae, *and* Streptococcus pneumoniae*. The paper disks containing the serial dilutions of atorvastatin, rosuvastatin, and simvastatin were placed on the lawn of bacteria along with their respective positive controls. After overnight incubation, the zones of inhibition around the disks were analyzed and tabulated.

Calculation of the minimum inhibitory concentration (MIC) using the broth dilution method

Preparation of the Antibiotic Dilution Range

The antimicrobial agent stock solutions at concentrations of 1000 μg/mL were prepared. Sterile 13 x 100 mm test tubes were used to conduct the test. The final twofold dilutions of the antimicrobial agent were prepared volumetrically in the broth. A minimum final volume of 0.1 mL of each dilution needed for the test was dispensed into every 96 wells of a standard tray.

Preparation of the Inoculum

The inoculum was prepared by making a direct broth suspension of isolated colonies selected from an 18- to the 24-hour agar plate. The suspension was adjusted to achieve turbidity equivalent to a 0.5 McFarland turbidity standard. This resulted in a suspension containing approximately 1 to 2 x 10^8^ colony-forming units (CFU)/mL for *Escherichia coli* American Type Culture Collection (ATCC®) 25922. The inoculum tube was compared with the 0.5 McFarland standard against a card with a white background and contrasting black lines.

Inoculation

Within 15 minutes after the inoculum was standardized as described above, 0.1 mL of the adjusted inoculum was added to each tube containing 0.1 mL of the antimicrobial agent in the dilution series and mixed. The growth control was the broth containing bacterial suspension, and the sterile control was normal saline.

Incubation

The inoculated tubes were incubated at 35 ± 2ºC for 16 to 20 hours in an ambient air incubator. After the incubation period, the optical density (OD) was measured and recorded. Each set of experiments was performed at least three times. From the OD obtained, the percentage growth inhibition was calculated using the formula as described by Bate et al. [18].

Statistics

Analysis was performed using GraphPad Prism software version 10.2.0. A one-way ANOVA test was used to determine if there was any statistical difference between the different statin and antibiotic groups. A one-sample t-test was used to observe the statistical difference between normal saline and statins. An unpaired t-test was used to determine the statistical difference between the statins.

## Results

Agar cup diffusion method

**Figure 1:**
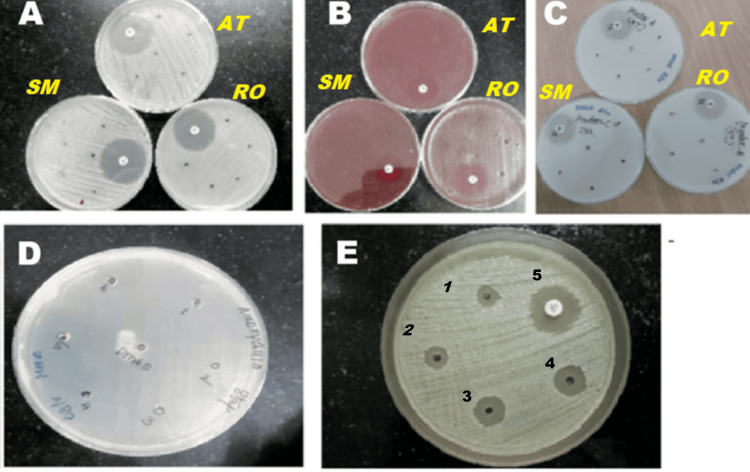
Agar cup diffusion method showing the zone of inhibition of atorvastatin, rosuvastatin, simvastatin in Gram-positive and Gram-negative bacteria using amoxicillin and gentamicin as standard, respectively (A) Zone of inhibition of atorvastatin (AT), rosuvastatin (RO), and simvastatin (SM) against *Staphylococcus aureus* in serial dilutions of 3.125 µg/ml, 6.25 µg/ml, 12.5 µg/ml, 25 µg/ml, and 50 µg/ml using amoxicillin as a standard. (B) Zone of inhibition of AT, RO, and SM against *Streptococcus pneumoniae* in serial dilutions of 3.125 µg/ml, 6.25 µg/ml, 12.5 µg/ml, 25 µg/ml, and 50 µg/ml using amoxicillin as a standard. (C) Zone of inhibition of AT, RO, and SM against *Klebsiella pneumoniae* in serial dilutions of 3.125 µg/ml, 6.25 µg/ml, 12.5 µg/ml, 25 µg/ml, and 50 µg/ml using gentamicin as a standard. (D) Zone of inhibition of amoxicillin in serial dilutions of 3.125 µg/ml, 6.25 µg/ml, 12.5 µg/ml, 25 µg/ml, and 50 µg/ml shown as markings 1, 2, 3, 4, and 5, respectively. (E) Zone of inhibition of gentamicin in serial dilutions of 3.125 µg/ml, 6.25 µg/ml, 12.5 µg/ml, 25 µg/ml, and 50 µg/ml shown as markings 1, 2, 3, 4, and 5, respectively.

Our results displayed a lack of antibacterial activity of all three statins, atorvastatin, rosuvastatin, and simvastatin, against all three bacterial strains isolated from sputum specimens: *Staphylococcus aureus, Klebsiella pneumoniae, *and *Streptococcus pneumoniae*, after overnight incubation by the agar cup method at concentrations of 3.125 µg/ml, 6.25 µg/ml, 12.5 µg/ml, 25 µg/ml, and 50 µg/ml, respectively, as shown in Figure 1. The zone of inhibition observed was less than 4 mm (resistant) for all the serial dilutions of atorvastatin, rosuvastatin, and simvastatin, as tabulated in Table 1. 

**Table 1:** The zone of inhibition of statins, gentamicin, and amoxicillin

Serial concentration	Amoxicillin (zone of inhibition)	Gentamicin (zone of inhibition)	Atorvastatin, rosuvastatin, and simvasatin (zone of inhibition)
3.125 µg/ml	>30 mm	10 mm	<4 mm (resistant)
6.25 µg/ml	>30 mm	12 mm	<4 mm
12.5 µg/ml	>30 mm	17 mm	<4 mm
25 µg/ml	>30 mm	18 mm	<4 mm
50 µg/ml	>30 mm	21 mm	<4 mm

Broth dilution method

**Figure 2:**
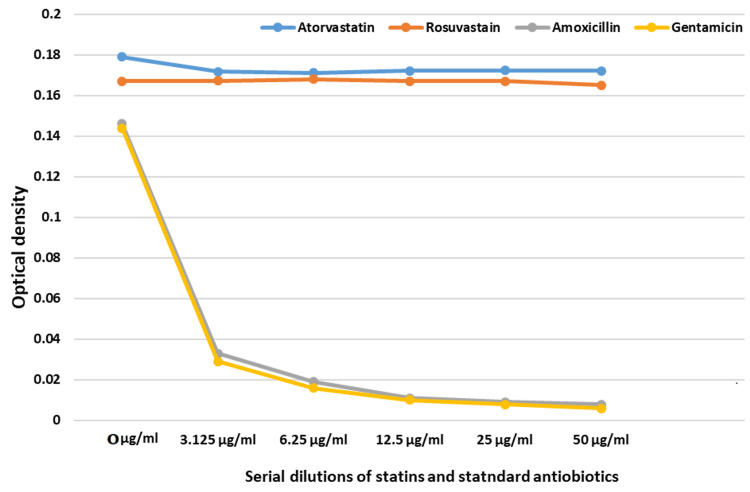
Optical density of statins and standard antibiotics in serial dilutions The observed initial optical density (OD) of broth containing only bacterial growth before the addition of atorvastatin is 0.1790. The OD after the addition of atorvastatin is 0.1718, 0.1712, 0.1721, 0.1724, and 0.1722 at 3.125 μg/ml, 6.25 μg/ml, 12.5 μg/ml, 25 μg/ml, and 50 μg/ml serial concentrations of the drug, respectively. The observed initial OD of broth containing only bacterial growth before the addition of rosuvastatin is 0.1670. The OD after the addition of  rosuvastatin is 0.1672, 0.1681, 0.1670, 0.1671, and 0.1650 at 3.125 μg/ml, 6.25 μg/ml, 12.5 μg/ml, 25 μg/ml, and 50 μg/ml serial concentrations of the drug, respectively. The observed initial OD of broth containing only bacterial growth before the addition of amoxicillin is 0.1460. The OD after the addition of amoxicillin is 0.0330, 0.0190, 0.0110, 0.0090, and 0.0080 at 3.125 μg/ml, 6.25 μg/ml, 12.5 μg/ml, 25 μg/ml, and 50 μg/ml serial concentrations of the drug, respectively. The observed initial OD of broth containing only bacterial growth before the addition of gentamicin is 0.1440. The OD after the addition of  amoxicillin is 0.0290, 0.0160, 0.0100, 0.0080, and 0.0060 at 3.125 μg/ml, 6.25 μg/ml, 12.5 μg/ml, 25 μg/ml and 50 μg/ml serial concentrations of the drug, respectively.

The OD of serial dilutions of atorvastatin, rosuvastatin, gentamicin, and amoxicillin as observed is shown in Figure 2.

From the OD, the percentage inhibition of bacterial growth was calculated using the following formula: (Control OD − (Sample OD/Control OD)) × 100. The minimum inhibitory concentration at which 90% of bacterial growth is inhibited (MIC90) is also tabulated as follows in Table 2. The ANOVA test showed amoxicillin and gentamicin to have high statistically significant microbial growth inhibitory activity (p-value < 0.005) compared to atorvastatin and rosuvastatin . However, one sample t-test showed atorvastatin to have statistically significant antimicrobial activity compared to normal saline. Rosuvastatin, on the other hand, did not have any statistically significant difference from normal saline. Unpaired t-test showed a statistically significant difference between the antimicrobial activity of atorvastatin and rosuvastatin (p-value < 0.0001).

**Table 2:** Optical density, percentage growth inhibition, and minimum inhibitory concentration of statins, gentamicin, and amoxicillin MIC: Minimum inhibitory concentration; SEM: standard error of mean; SD: standard deviation; N: number of serial drug concentrations Positive control is bacterial growth without treatment with statins and standard antimicrobials amoxicillin and gentamicin. Negative control is normal saline. -Not applicable. *Amoxicillin and gentamicin showed statistically significant microbial growth inhibitory activity (p-value < 0.0001) compared to atorvastatin and rosuvastatin. ^#^Atorvastatin showed a statistically significant microbial growth inhibitory activity (p-value < 0.0001) compared to normal saline. Rosuvastatin showed no statistically significant microbial growth inhibitory activity (p-value > 0.05) compared to normal saline.

Concentration	Atorvastatin optical density	Rosuvastatin optical density	Amoxicillin optical density	Gentamicin optical density	Atorvastatin		% Growth inhibition rosuvastatin		% Growth inhibition amoxicillin		% Growth inhibition gentamicin		MIC 90 (mg/ml) of amoxicillin	MIC 90 (mg/ml)) of gentamicin
					% Growth inhibition	Statistical parameters	% Growth inhibition	Statistical parameters	% Growth inhibition	Statistical parameters	% Growth inhibition	Statistical parameters		
3.125 μg/ml	0.1718	0.1672	0.0330	0.0290	4.02	Mean: 3.928	-0.119	Mean: 0.07440	77.39	Mean: 89.032	79.86	Mean: 90.412	0.125 mg/ml	0.62 mg/ml
6.25 μg/ml	0.1712	0.1681	0.0190	0.0160	4.35	SEM: 0.119	-0.65%	SEM: 0.30434	86.98	SEM: 3.197	88.88	SEM: 2.883		
12.5 μg/ml	0.1721	0.1670	0.0110	0.0100	3.8	N - 5	0%	N - 5	92.46	N - 5	93.05	N - 5		
25 μg/ml	0.1724	0.1671	0.0090	0.0080	3.68	SD: 0.2662	-0.059	SD: 0.68053	93.83	SD: 7.148	94.44	SD: 6.4465		
50 μg/ml	0.1722	0.1650	0.0080	0.0060	3.79	p-value: 0.0001^#^	1.2	p-value: 0.8189	94.5	p-value: <0.0001^*^	95.83	p-value: <0.0001^*^		
Positive control	0.1790	0.1670	0.1460	0.1440	-	-	-	-	-	-	-	-		
Negative control	0.0030	0.0030	0.0030	0.0030	-	-	-	-	-	-	-	-		

## Discussion

Many studies suggest the antibacterial activity of statins and their role in the prevention and treatment of many infections. Analogously, a few studies have also suggested its beneficial role in reducing mortality and preventing infections in people with pneumonia and other infections of the respiratory tract. Nevertheless, our study showed that atorvastatin, rosuvastatin, and simvastatin lacked antibacterial activity in all three bacterial strains, *Staphylococcus aureus, Klebsiella pneumoniae, *and *Streptococcus pneumoniae*, isolated from sputum specimens, after overnight incubation by the agar cup method at concentrations of 3.125 μg/ml, 6.25 μg/ml, 12.5 μg/ml, 25 μg/ml, and 50 μg/ml, respectively.

The minimum inhibitory concentration calculated using the broth dilution method also showed no significant antibacterial activity of atorvastatin and rosuvastatin even at the highest concentration of 50 mcg/ml compared to standards amoxicillin and gentamicin.

This finding is, however, in concurrence with studies conducted by Bergman et al. and Kornelsen et al., who also testified to the lack of antibacterial activity of statins against various bacterial strains [15,19].

However, atorvastatin showed statistically significant antimicrobial activity compared to normal saline and also showed a statistically significant difference from the antimicrobial activity of rosuvastatin. This nevertheless cannot be considered clinically significant as the antimicrobial activity shown by atorvastatin does not correspond to the dose of the drug used.

The explanation for the potent antibacterial activity of statins, as revealed by numerous other studies contrary to ours, is still not clear. The possible reasons for the discordance might be due to the use of very high doses of statins above the normal physiologically acceptable therapeutic plasma level. According to the studies conducted by Jerwood et al. and Masadeh et al., statins possessed antibacterial activity but at a dose greater than the normal acceptable therapeutic serum concentration of statins [20, 21]. The antibacterial effect of statins at these very high doses is practically not going to benefit the patient in any way, as statins are themselves associated with their adverse effects like hepatic damage and myopathy. We have restricted the dose of statins to a maximum of 50 mcg/ml in our study. Genetic heterogeneity among bacterial strains used for testing might also be the reason for the dissimilarity of results.

Incorrect epidemiological data due to publication bias also plays a major role, as many genuine scientific studies may fail to get published due to negative results. This will create a strong false belief among researchers with each positive publication.

Limitations of the study

Since this is an in vitro study, the antibacterial effect of statins on long-term intake among patients is unknown. The synergistic activity of statins with other antimicrobials has not been studied. The role of statins in the prevention of bacterial infections is also not known. Hence, there is a need for randomized controlled trials of statin use among patients with respiratory tract infections, which could help us come to a clear-cut conclusion.

## Conclusions

Atorvastatin, rosuvastatin, and simvastatin lacked antibacterial activity against all three bacterial strains isolated from sputum specimens: *Staphylococcus aureus, Klebsiella pneumoniae,* and *Streptococcus pneumoniae *at normal therapeutic plasma concentrations. Though atorvastatin had negligible antimicrobial activity when compared to normal saline, it is not considered clinically significant as the microbial effect exerted by it is far inferior to standard antimicrobials like amoxicillin and gentamicin. Hence, the use of statins as antimicrobial drugs for respiratory tract infections has limited applications. However, the antimicrobial effect of statins at higher concentrations is still debatable and requires further research.
